# Phenotypic and genetic characteristics of a Dutch cohort of patients with X-linked osteoporosis due to *PLS3* genetic variants

**DOI:** 10.1093/jbmrpl/ziaf046

**Published:** 2025-04-24

**Authors:** Zografia Zervou, Hennie T Bruggenwirth, Serwet Demirdas, M Carola Zillikens

**Affiliations:** Department of Internal Medicine, Erasmus MC Bone Center, Erasmus University Medical Center, Dr. Molewaterplein 40, 3015 GD, Rotterdam, The Netherlands; Department of Clinical Genetics, Erasmus MC, Erasmus University Medical Center, Dr. Molewaterplein 40, 3015 GD, Rotterdam, The Netherlands; Department of Clinical Genetics, Erasmus MC, Erasmus University Medical Center, Dr. Molewaterplein 40, 3015 GD, Rotterdam, The Netherlands; Department of Internal Medicine, Erasmus MC Bone Center, Erasmus University Medical Center, Dr. Molewaterplein 40, 3015 GD, Rotterdam, The Netherlands

**Keywords:** X-linked osteoporosis, *PLS3*, phenotype, genotype, plastin-3

## Abstract

X-linked osteoporosis, caused by plastin 3 (*PLS3*) genetic variants, is a rare disease, characterized by low BMD and early-onset fractures, primarily affecting men. Our aim was to further elucidate the phenotypic characteristics, including sex-differences and genotype–phenotype analysis, in individuals with *PLS3* variants. Our cohort comprises of 28 patients from 11 families, 18 men and 10 women, with a different *PLS3* variant in each family. Demographic, clinical, and genetic features, imaging and laboratory tests, and treatment details were retrospectively reviewed. Men, (median age 47.0 y), demonstrated low Z-scores of the lumbar spine (−2.8 ± 1.7) and femoral neck (−1.7, IQR: −2.9-0.8). Most women (median age 49.5 y) had normal BMD, two had osteoporosis and one osteopenia. Moreover, men experienced a higher total number of fractures than women (men: 12.0, IQR: 6.7, 18.5, women: 2.0, IQR: 0.7, 5.2). Within one large family (*n* = 10) there was considerable heterogeneity regarding BMD and fractures, which might be explained by differences in factors like physical exercise (PE) or in (poly) genetic background. Extra-skeletal characteristics such as (mild) blue discoloration of the sclerae (men: 33.3%, women: 30.0%), joint hypermobility (44.4%, 70.0%) and skin hyperlaxity (50.0%, 20.0%) were observed. No relation was found between types and locations of variants and various clinical endpoints in men, using data from our cohort and the literature. Regarding treatment, all men and 40% of women received bone-active therapy, mostly oral bisphosphonates. Adult men demonstrated a 16.6% mean increase in the BMD of the lumbar spine (*p* = .03), after a median treatment duration of 6 y. In summary, this is so far the largest study of patients with X-linked osteoporosis, including an extensive genotype–phenotype analysis. A potential protective role of increasing weight-bearing PE in osteoporosis severity, as well as effects of penetrance, genetic background, or other environmental or lifestyle factors, need further study.

## Introduction

Osteoporosis is a complex metabolic bone disorder with high worldwide prevalence affecting millions of people. It is defined by low bone density and disruption of the bone architecture resulting in bone fragility and fractures.^1^^,^^2^ In addition to environmental and hormonal factors, genetic predisposition plays an important role in the development of bone fragility.^3^ There are several rare monogenic forms of bone fragility, the most common one being osteogenesis imperfecta (OI) which is mostly caused by dysregulation of *COL1A1* and *COL1A2* genes, involved in the production of collagen type I.^4^ Collagen type I is vital for the bone extracellular matrix, providing strength and flexibility through its triple helical structure.^5^ Patients with OI develop bone fragility that leads to fractures and deformities. Extra-skeletal characteristics such as hearing loss, dental abnormalities, blue sclerae, respiratory and cardiovascular complications, are also seen in OI, as collagen type I is also present in other tissues.^6^ Apart from OI there are several forms of monogenic bone diseases with early onset osteoporosis.

One of these rare forms of hereditary osteoporosis was first described in 2013: X-linked osteoporosis, caused by (likely) pathogenic variants in the *PLS3* (OMIM number: 300131) gene which codes for plastin 3 (PLS3), an F-actin binding and bundling protein which is widely expressed in solid tissues.^7^ This X-linked form of primary osteoporosis is characterized by low BMD, frequent and early onset peripheral and vertebral compression fractures (VCFs), sometimes resulting in severe thoracic kyphosis. Due to its X-chromosomal inheritance pattern, hemizygous males are typically more severely affected than heterozygous females.^8–13^ Heterozygous females present with a variable phenotype ranging from no fractures and normal BMD to skeletal fragility with few or multiple fractures.^7^^,^^9^^,^^11–18^ Extra-skeletal features, resembling those in OI, have also been observed including blue sclerae, joint hyperlaxity, and hearing loss.^10–13^^,^^15^^,^^17^^,^^19–22^ The mechanisms leading to those features remain unclear, as *PLS3* is not directly involved in collagen type I formation. Interestingly, autism spectrum disease has also been reported before in patients with OI and *PLS3* related X-linked osteoporosis,^22^^,^^23^ but this relation has not been structurally investigated.

While the role of *PLS3* in bone fragility is yet unknown, one theory presumes that *PLS3* genetic variants lead to decreased mechanosensing of osteocytes.^7^ The elevated expression of *PLS3* in chicken osteocyte dendrites, especially during dendrite formation, is providing support for this theory.^24^^,^^25^ Another study suggested a role for *PLS3* in bone mineralization. The latter is based on the findings of bone biopsies of 2 adults and 5 paediatric male patients and 1 adult female patient where a low bone turnover with reduced osteoblast and osteoclast numbers and reduced matrix mineralization^8^^,^^10^^,^^26^ was seen, although serum bone turnover markers were mostly normal.^8^^,^^10^ Disturbed bone mineralization could lead to osteomalacia, which could also influence mechanosensing. Osteomalacia is a pathological bone condition consisting in a deficient primary mineralization of the matrix, leading to an accumulation of osteoid tissue and reduced bone mechanical strength. The amounts, properties and organization of bone constituents at tissue level, are known to influence its mechanical properties.

However, the comprehension of the clinical and genetic spectrum as well as the natural history of the disease and best treatment options remains limited. To date, only 47 families have been described with 47 different genetic variants.^7–22^^,^^27–29^

Our aim was to study the phenotypic characteristics of X-linked osteoporosis due to *PLS3* genetic variants in the, so far, largest clinical cohort, the differences between the two sexes and the effect of medical treatment on the course of the disease. Furthermore, we examined the relation between different types and location of the genetic variants of *PLS3* and the phenotype.

## Materials and methods

### Study population

This single-center retrospective observational study was conducted at the Bone Center of the Erasmus Medical Center, the Netherlands. Study participants were included if a genetic variant in *PLS3* was found. In total, 28 patients: 18 men and 10 women from 11 families were included (Table S1). Data were collected from January 2020 until August 2024. Genetic analysis was performed, in most of the patients, through next generation sequencing (NGS), followed by analysis of variants implicated in OI and other monogenic bone diseases. The study was approved by the Ethics Committee of the Erasmus Medical Center and a written informed consent was obtained from the participants and/or their parents (MEC-2020-0737).

**Table 1:** Demographic characteristics.

	**Men (*n* = 18)**	**Women (*n* = 10)**
**Age (yr)**	47.0 (25.0-59.0)	49.5 (31.7-62.5)
**Age at first fracture (yr)**	8.0 (6.0-14.8)	9.0 (1.0-37.5)
**Age at genetic diagnosis (yr)**	46.5 (17.0-53.5)	41.0 (22.0-43.0)
**Age at starting treatment (yr)**	29.0 (11.0-45.5)	44.0 (22.5-64.5)
**Height of adults at first visit (cm)**	181.0 (175.0-183.0)	166.0 (160.0-172.5)
**BMI of adults at first visit (kg)**	24.7 (23.9- 29.2)	25.2 (22.7-35.2)

Data are displayed as median with 25th and 75th percentile in parentheses.

### Clinical data collection

Demographic data, clinical evaluations, radiographic assessments, and biochemical analyses were retrospectively collected from patients’ electronic files. The data were obtained at the Erasmus Medical Centre or, previously, in other hospitals as part of standard clinical practice. The following demographics were analyzed: age (at entry in the study database), age at first fracture, age at genetic diagnosis, age at starting treatment, height (at first visit), BMI (at first visit) and degree of physical activity (from childhood until the most recent visit during data collection), although no standardized questionnaire was used. Age at diagnosis was considered the age at which the presence of a *PLS3* genetic variant was established through genetic analysis. Furthermore, the following clinical features were included and investigated in the study: BMD at the lumbar spine and femoral neck and their respective Z-scores, the number of fractures (non-vertebral, vertebral, and finger/toe fractures), the use of bone-active therapy and the effect on BMD, the age at starting treatment, the duration of treatment and the prevalence of extra-skeletal traits including mild blue discoloration of the sclerae, joint hypermobility, skin hyperlaxity, and hearing loss. Additionally, we have evaluated the medical history and the patient electronic files for the presence of autism spectrum disease, which, as mentioned, has been previously reported in patients with *PLS3* genetic variants.^22^^,^^23^ All patients were screened, as part of the clinical care, for the presence of prevalent vertebral fractures through X-rays or vertebral fracture assessment (VFA), according to the method of Genant et al. and classified according to vertebral height loss (grade 1: 20%–25%; grade 2: 25%–40%; grade 3: ≥40%).^30^^,^^31^ We assessed the presence of joint hypermobility by physical examination in which we evaluated the following: whether there was overextension of knees and elbows, whether the thumb could be brought to the forearm and whether the patient standing with knees straight and legs together could touch the floor with flat hands. Skin hyperlaxity was evaluated by stretching a skinfold on the back, by pinching the skin between the thumb and middle finger and pulling the skinfold, until resistance was present. All patients were asked about problems related to their hearing ability. BMD measurements by DXA were for most of the patients performed using GE Lunar Prodigy Advance equipment (H8950AN enCORE, Version 17 SP4); Z-scores were calculated using equipment-specific and age- and sex adjusted reference data combination of National Health and Nutrition Examination Survey (NHANES) (age 20-30) and Lunar (age 20-40), and T-scores using equipment-specific reference data for white and age matched females.

### Genotype–phenotype connection

Genetic variants were classified according to the American College of Medical Genetics (ACMG) guidelines^32^ (Table S1). To analyze the relationship between genotype and phenotype, data from literature combined with data from our cohort on genetic and clinical aspects of X-linked osteoporosis were collected.^7–12^^,^^14–22^^,^^27–29^^,^^33^ When looking at genotype–phenotype correlations, results from female carriers were excluded due to their variable phenotype, probably caused by (skewed) X-inactivation. In total, data of 72 men are reported. The age at first fracture, the Z-score of the lumbar spine and femoral neck, the total number of fractures, the total number of vertebral and peripheral fractures, the use of bisphosphonates, duration of treatment, the existence of (mild) blue discoloration of the sclera and joint hypermobility were compared across different types of genetic variants and their respective locations. The genetic variants were categorized into two types among individuals with *PLS3* genetic variants and not only index patients: frameshift/nonsense/complete deletion of the gene (*n* = 46), missense/in-frame insertion/splice-site/intra-gene deletion/duplication (*n* = 26), according to the presumed effect on the protein production, and the locations were divided into 5 functional domains: EF1 (*n* = 7), EF2 (*n* = 8), Linker (*n* = 3), ABD1 (*n* = 22), and ABD2 (*n* = 18) (Figure 3).

### Statistical analysis

Statistical analysis was conducted by SPSS Statistics 28 (IBM Corporation). Normality of the data was assessed using Kolmogorov–Smirnov, Shapiro–Wilk tests, and visually evaluating histograms. Descriptive data are reported as mean ± SD or median (IQR) depending on their distribution. Paired or unpaired *t* tests were used to compare two groups. Unpaired *t*-test, one way ANOVA, Mann–Whitney U, Kruskal–Wallis, or Fisher exact test were applied to analyze phenotypic differences between the different types of genetic variants and the location of each mutation. The significance level was set at α = .05.

## Results

### Demographic characteristics

The demographics and clinical characteristics of patients from our own cohort are shown in Table 1. Both male (*n* = 18) and female *(n* = 10) patients had an early onset of the fractures (8 and 9 y of age, respectively). Men started treatment at an earlier age than women (29 and 44 y, respectively). Median height of adult patients was normal at first visit compared to the average height of adult Dutch males (183.8 ± 7.1 cm) and females (170.7 ± 6.3 cm).^34^ Regarding family history, 14 men reported a positive maternal history; in 9 cases a positive genetic test was documented, in 1 case the mother was obligate carrier (and in 4 cases there was a fracture history and/or low BMD in the mothers). The following was reported for women: in two cases a positive paternal history based on a positive genetic test, in two cases a positive maternal history based on genetic test, one patient reported fracture history and/or low BMD in family history and in two cases mother was obligate carrier. This concerns two sisters with affected children, not genetically tested, one of them reported above in the family history of men.

### Genetic characteristics

Genetic variants that could result in the absence of protein production due to nonsense-mediated decay, were predominantly seen: 5 families had a frameshift deletion and 2 had a nonsense variant. Furthermore, an in-frame insertion was reported in one family, a missense in one family and a splice-site variant in 2 families (family 9 and 11, Table S1). Six of the genetic variants reported in our cohort are novel and have not been described before in 1000 Genomes, ExAC, PubMed, ESP6500, ClinVar, and HGMD databases (Table S1). Additionally, genetic variants were classified according to the ACMG guidelines: 4 families had a pathogenic variant (class 5), 4 families a likely pathogenic variant (class 4), and 3 families a variant of unknown clinical significance (class 3) (Table S1).

### Clinical characteristics

**Figure 1:**
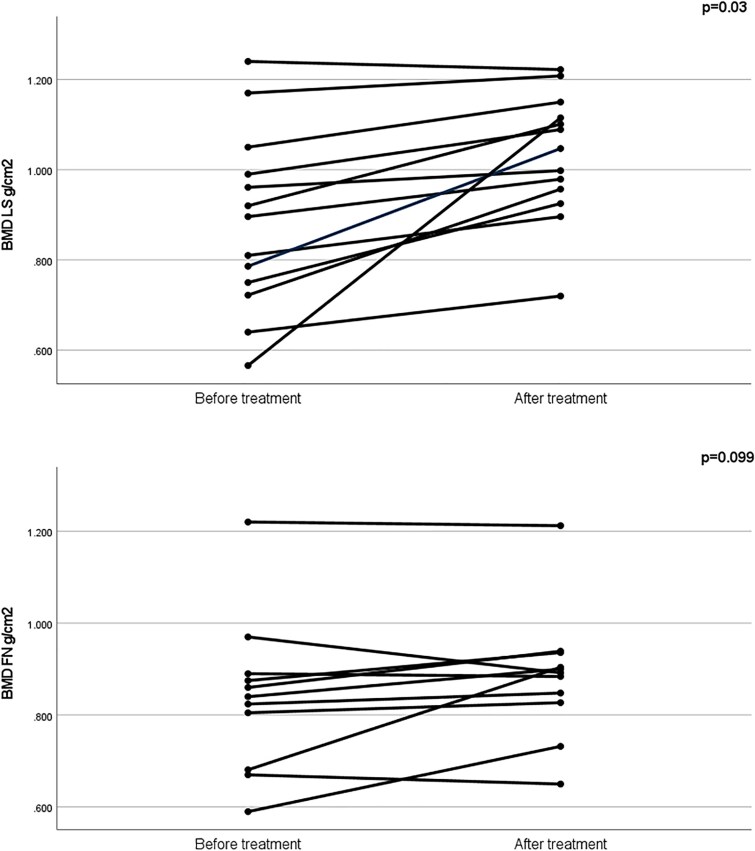
Follow-up BMD of adult males before and after treatment with bisphosphonates. Median duration of treatment between measurements of adult males was 6 years. The patient exhibiting the most remarkable increase in LS BMD, received bisphosphonates for 13 years since age 47. *p*-values refer to the outcome of the paired *t*-test between the two values for LS and FN BMD.

Men demonstrated lower BMD and higher number of non-vertebral and vertebral fractures than females (Table 2). Median Z-scores at first visit, at our center, were low among men for both the lumbar spine the femoral neck and normal for women (Table 2). Additionally, 88.8% of men and 70.0% of women had non-vertebral fractures before the age of 18 y. Furthermore, 33.3% of men and 20.0% of women had ≥2 finger or toe fractures. Regarding vertebral fractures, the majority (88.8%) of men had vertebral fractures (grade 1–3) and 50.0% of them had one or more grade 3 vertebral fracture. A high proportion of men (83.3%) developed vertebral fractures before the age of 50 y (Table 2). All men and 50% of the women received bone active medication. Specifically, 88.8% of men and 20.0% of women received oral bisphosphonates (BPs), and 50.0% of men were treated with oral and intravenous BPs sequentially (Table 2). Two women (20%) received intravenous BPs at different institutions, off-label, and due to fatigue that was considered by their physicians to be possibly related to the *PLS3* variants. In addition to BPs, three men and two women had another form of bone active medication (Table 2). Regarding men, the first patient switched from BPs to denosumab due to new vertebral fractures. The second patient switched from BPs to teriparatide because of progression of vertebral fractures and he was, subsequently, transitioned to denosumab. The third patient started teriparatide after 5 y without treatment due to progression of two, already existing, vertebral fractures. Regarding women, two sisters received bone anabolic agents. The first one received teriparatide because of multiple new vertebral fractures and the second one received sequentially zoledronate, teriparatide, zoledronate, and romosozumab. The first switch to bone anabolic agents was due to new vertebral fractures and the second due to BMD decrease. Adult men who started BPs in adulthood, demonstrated a 16.6% mean increase in LS BMD (*p* = .03), after a median duration of treatment 6 y (Figure 1). Additionally, new vertebral fractures occurred in only 3 men after initiating therapy due to non-compliance in 2 of them, including the patient mentioned above who missed a dose of denosumab. The other patient experienced one new grade 2 vertebral fracture with inconsistent risedronate intake. Physical examination revealed a high prevalence of extra-skeletal characteristics, in a mild form, among our patients. Mild blue discoloration of the sclera was seen in 33.3% of men and in 30.0% of women, joint hypermobility in 44.4% of men and 70.0% of women and increased skin laxity in 50.0% of men and in 20.0% of women (Table 3). No hearing-loss was reported.

**Table 2:** Clinical characteristics of individuals with *PLS3* variants.

	**Men (*n* = 18)**	**Women (*n* = 10)**
**Total number of fractures** ^a^	12.0 (6.7, 18.5)	2.0 (0.7, 5.2)
**Total number of vertebral fractures** ^a^	4.5 (2.0, 7.7)	0.0 (0.0, 1.0)
**Total number of non-vertebral fractures** ^a^	4.0 (2.0, 9.0)	1.0 (0.0, 2.2)
**Presence ≥2 finger/toe fractures, % (*n*)**	33.3 (6)	20.0 (2)
**Non-vertebral fractures <18y, % (*n*)**	88.8 (16)	70.0 (7)
**Presence of VCFs, % (*n*)**	88.8 (16)	20.0 (2)
**VCFs <50 y, % (*n*)**	83.3 (15)	0.0 (0)
**Grade 1 VCFs, % (*n*)** ^b^	66.7 (12)	0.0 (0)
**Grade 2 VCFs, % (*n*)** ^b^	61.1 (11)	20.0 (2)
**Grade 3 VCFs, % (*n*)** ^b^	50.0 (9)	20.0 (2)
**LS BMD (g/cm** ^**2**^ **) at first visit**	0.73 ± 0.2	1.06 (0.94, 1.21)^a^
**LS Z-scores at first visit**	−2.8 ± 1.7	−0.9 (−2.1, 0.2)^a^
**FN BMD (g/cm** ^**2**^ **) at first visit** ^a^	0.78 (0.67, 0.87)	0.87 (0.80, 0.98)
**FN Z-scores at first visit** ^a^	−1.7 (−2.9, −0.8)	−0.5 (−1.8, −0.3)
**Use of bone active medication, % (*n*)**	100.0 (18)	50.0 (5)
**Use of oral BPs, % (*n*)**	88.8 (16)	20.0 (2)
**Use of intravenous BPs, % (*n*)**	61.1 (11)	30.0 (3)
**Use of both types of BPs sequentially, % (*n*)**	50.0 (9)	0.0 (0)
**Use of denosumab, % (*n*)**	11.1 (2)	0.0 (0)
**Use of teriparatide, % (*n*)**	11.1 (2)	10.0 (1)
**Use or romosozumab, % (*n*)**	0.0 (0)	20.0 (2)
**VCFs after initiation of therapy, % (*n*)** ^c^	16.7 (3)	25.0 (1)
**Non-vertebral fractures after initiation of therapy, % (*n*)** ^c^	38.9 (7)	0.0 (0)
**Duration of therapy (yr)** ^a^	7.5 (4.7, 14.2)	1.0 (1.0, 13.5)
**Duration of therapy adults (yr)** ^a^	11.0 (5.0, 16.0)	4.5 (1.0, 16.2)
**Dairy intake servings/d** ^a^	2.0 (2.0, 4.0)	3.5 (2.0, 5.2)
**Use of calcium supplements, % (*n*)**	38.9 (7)	0.0 (0)
**Use of vitamin D supplements, % (*n*)**	94.4 (18)	60.0 (6)

a Data are displayed as median with 25th and 75th percentile in parentheses.

b According to Genant et al. classification.^30^^,^^31^

c First vertebral fracture occurred after a median duration of treatment of 7.5 y (IQR:5-10) and first non-vertebral after 6 y (IQR: 2-10.5).

Abbreviations: BPs, bisphosphonates; VCFs, vertebral compression fractures.

**Table 3:** Presence of extra-skeletal characteristics in *PLS3* individuals.

	**Men (*n* = 18)**	**Women (*n* = 10)**
**(Mild) blue discoloration of the sclerae, % (*n*)**	33.3 (6)	30.0 (3)
**Joint hypermobility, % (*n*)**	44.4 (8)	70.0 (7)
**Skin hyperlaxity, % (*n*)**	50.0 (9)	20.0 (2)
**Autism spectrum disease, % (*n*)**	11.1 (2)	10.0 (1)
**Epilepsy, % (*n*)**	5.5 (1)	0.0 (0)

### Genotype–phenotype relation

**Figure 2:**
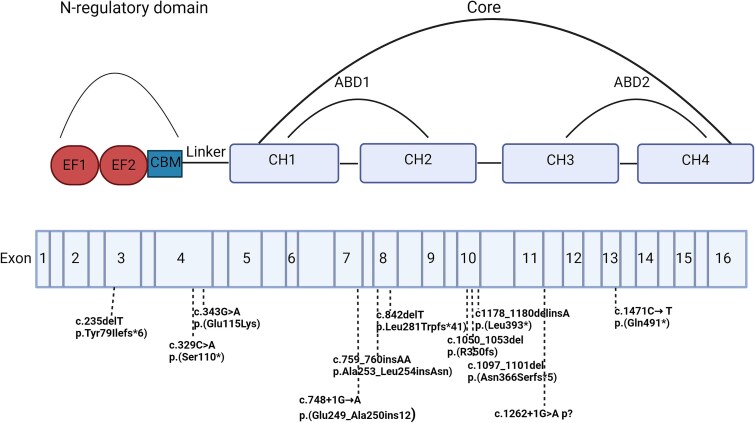
Plastin-3 protein and the location of the variants in this cohort. Created with BioRender.com. Plastin-3 protein consists of an N-regulatory domain, a linker and a core consisting of two actin-binding domains. Summary of all the variants described in the literature recently published.^29^ The structure of the gene is illustrated below the protein. The figure displays the exons and introns of the *PLS3* gene, along with the genetic specifics and the positions of the reported genetic variants. Abbreviations: ABD, actin-binding domain; CBM, calmodulin-binding motif; CH, calponin-homology domain; EF, EF-hand motifs.

**Figure 3:**
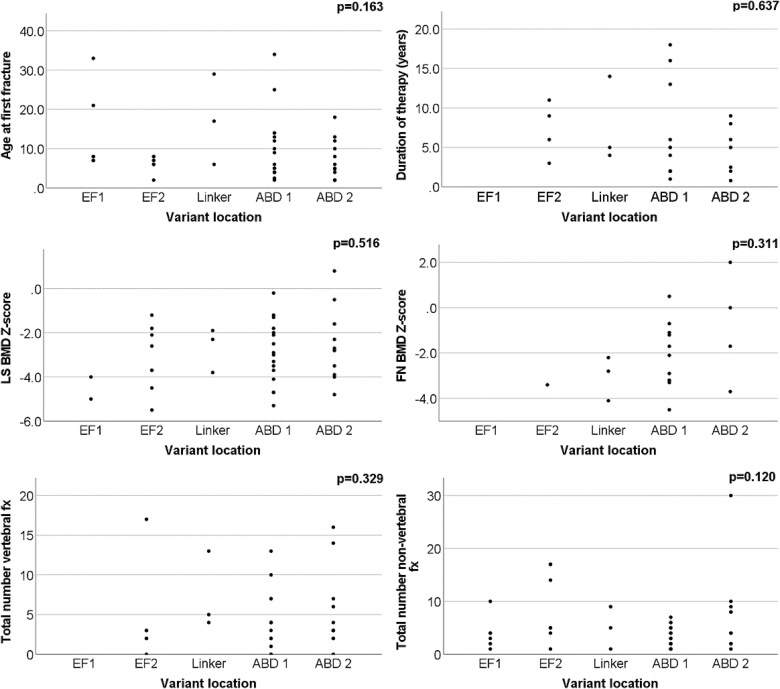
Relation of the variant location with the age at first fracture, duration of therapy, LS and FN Z-score and the total number of vertebral and non-vertebral fractures. No relation was found between types and locations of genetic variants of 72 men and various clinical endpoints. *p*-values refer to differences between all groups and were calculated with ANOVA for normally distributed variables or Kruskal–Wallis for non-normally distributed data. Abbreviation: fx, fractures.

In a total of 72 men, both from our study and the literature, no relation between genotype and phenotype was identified. Specifically, no relation was found between the different variant types and the age at first fracture, the Z-score of the lumbar spine, the Z-score of the femoral neck, the total number of fractures, the total number of vertebral fractures and non-vertebral fractures, the use of BPs, the duration of treatment, the presence of blue/light blue sclera and hypermobility of joints. Consistent with our findings about the different variant types, we did not find any relation between the different variant locations and the above-mentioned parameters (Figures 2 and 3).

### Intra-familial phenotypic differences and potential effects of physical activity

**Figure 4:**
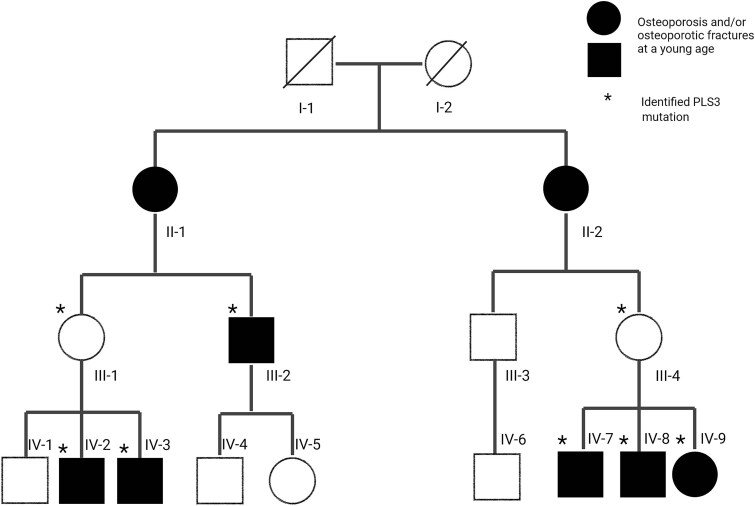
Family 7 with *PLS3* genetic variant. Created with BioRender.com. Remarkable differences in the clinical severity were seen among members of the same family, harboring the same variant. Differences in physical activity were observed among men, while older age, immobilization and comorbidity were noticed in women with a more severe phenotype.

Intra-familial differences in the phenotype were observed among male and female individuals of a previously reported family (family 7, Table S1, Figure 4). In particular, individuals IV-2 and IV-3 demonstrated a milder phenotype characterized by higher Z-scores in the lumbar spine (Z-score: −1.8SD, −2.1SD, respectively) compared to IV-7 and IV-8 (Z-score: −4, 5, −3, 7, respectively) (Table S2). Moreover, these individuals IV-2, IV-3 experienced fewer fractures (5, 7, respectively) than IV-7 and IV-8 (20, 22) and received treatment for a shorter duration (IV-2, IV-3 for 6 and 3 y, respectively, IV-7, IV-8 for 11 and 9 , respectively) (Table S2). A noteworthy difference in their lifestyle was the type of sport activities they were practicing. Individuals IV2-3 were playing competitive baseball, starting at age 6 and 7 respectively, for more than 3 hour per week for at least 11 y (Table S2), whereas individuals IV-7 and IV-8 were mostly engaged in competitive swimming during childhood. Phenotype differences were also seen among women in this family. Advanced age, immobilization and comorbidity were noticed in women with a more severe phenotype. Specifically, female individual II-1 was, at age 61, diagnosed with familial osteoporosis and a prevalent vertebral fracture. She received risedronate, but over the years she was lost to follow-up. Recently, at age 79, she suffered from severe back pain, after heavy coughing. Her medical history indicated that there was a back injury sustained 1 y ago, but no imaging was conducted at that time. X-ray revealed four new vertebral fractures, three mild and one severe. Due to the pain, she became mostly wheelchair bound. Her sister, individual II-2, 76 y old, developed multiple mild and severe vertebral fractures at age 72. These fractures occurred after 1 y of immobilization due to transverse myelitis. Moreover, her granddaughter, individual IV-9, 22 y old, has osteopenia and suffered multiple peripheral fractures in childhood all of them after adequate trauma (Table S2). Her medical history revealed Langerhans histiocytosis at age 1, for which she received chemotherapy for 1.5 y. In contrast, female individuals III-1 and III-4, both post-menopausal, have a mild phenotype with higher Z-scores of the lumbar spine (Z-score: 0.5 and 0.0, respectively), no vertebral fractures and few peripheral fractures (2 for each of them) (Table S2).

We also noticed that two elderly male patients, after multiple fractures, had stable disease for several years without a decrease in BMD and hardly any fractures, without medication. Both reported on follow-up visits that they had change their physical activities, quite dramatically, after being counseled about this at the out-patient clinic (family 2 and 6, Table S1).

## Discussion

The aim of this study was to further study the phenotypic characteristics of individuals with *PLS3* genetic variants and investigate sex-differences and genotype–phenotype associations. Consistent with previous publications, men exhibited a more severe phenotype than women and they were prescribed medical treatment more often and for longer duration. We found no genotype–phenotype relationships in men for different variants and locations with any of the phenotypic traits based on our own and literature data. Interestingly, there were some quite remarkable intra-familial differences in disease severity, especially in the largest family (*n* = 10). The latter underlines the difficulty of finding genotype–phenotype relations, as notable differences were observed within one family harboring the same variant.

Sequence variants in *PLS3* have so far been described in 47 families as the underlying cause for early onset osteoporosis and bone fragility.^7–22^^,^^27^^,^^28^ Here, we describe 28 patients seen at our Bone Center from 11 different families with 11 genetic variants in the *PLS3* gene. Six of those variants are novel. To the best of our knowledge, this is the largest cohort reported thus far. The results of our study provide more insight into the differences between the sexes and family members. Men and women experienced their first fracture at a young age, but males developed a more severe skeletal phenotype, including a higher number of vertebral and non-vertebral fractures and lower Z-scores both at the lumbar spine and femoral neck. BPs appear to be effective in increasing BMD and reducing the fracture rate in most of the patients, as demonstrated by previous research as well,^8^^,^^10^^,^^12–15^^,^^17^^,^^20^^,^^22^^,^^28^^,^^35^ although some patients needed anabolic therapy for disease progression. We observed mild extra-skeletal characteristics, as are typically described in OI, in several of our patients, including a slight blue discoloration of the sclerae, mild joint hypermobility and skin hyperlaxity. In OI patients with genetic variants in *COL1A1* and *COL1A2* genes this is^36^ attributed to a decrease in collagen thickness or composition.^37^ It is currently unclear why patients with *PLS3* variants would exhibit extra-skeletal manifestations and if there is a relation of *PLS3* with collagen metabolism or matrix composition, but similar findings have previously been documented in other individuals with *PLS3* genetic variations.^10–13^^,^^15^^,^^17^^,^^19–22^ Moreover, in one patient in our cohort, there was a description of a decreased amount of collagen type I in his skin biopsy taken in childhood before the first publication of PLS3 related osteoporosis (data not shown). In addition, we noticed the presence of autism and epilepsy among our patients. Both epilepsy and autism have been reported before in three studies of patients with OI and *PLS3* genetic variants, suggesting but not proving that neurological abnormalities may be linked to genetic causes of bone fragility.^12^^,^^22^^,^^23^ More research into the prevalence of these disorders, in larger cohorts of patients with monogenic bone disorders, is needed.

So far, the majority of the genetic variants reported in *PLS3* are either frameshift or nonsense variants, which result in nonsense-mediated mRNA decay. There have also been cases of large intragenic deletions or duplications, which can disrupt the structure of the gene, as well as splice-site genetic variants that could lead to truncated proteins. Previous research by Wu et al., among 43 males harboring *PLS3* genetic variants, could not demonstrate a relation between the type of the genetic variant and the BMD.^28^ It was hypothesized that all types of genetic variants could lead to loss-of-function. We performed a genotype–phenotype analysis with more clinical endpoints, in 72 men, including data from Wu et al. and other recent studies.^13^^,^^29^ Additionally, we also studied a relation with the location of the genetic variants, but we also failed to demonstrate any relations. Furthermore, we present differences in the phenotype of male individuals harboring the same genetic variant. Consistent with the absence of a genotype–phenotype association we observed that in one large family there were quite remarkable differences in clinical severity. These differences may be explained by differences in penetrance, other lifestyle factors such as physical activity or differences in genetic background such as in polygenic risk score for low BMD.

Our study has several limitations. Firstly, no standardized test was used to measure joint hypermobility and skin laxity. Moreover, serial-BMD was measured in many patients, on different machines. Another limitation is the lack of availability of data for reliable prospective analysis on the effect of medications. Additionally, no physical activity questionnaire or measurement of bone strength was available. Lastly, no-functional studies were conducted to evaluate the production of the PLS3 protein and the possible phenotypic. Further research, with detailed physical activity questionnaires in relation to the severity of the bone phenotype among individuals with *PLS3* genetic variants, is needed to confirm and extend our findings of a potential relationship with physical activity. Measurement of muscle mass and strength might in future studies also be relevant, both as a marker of physical activity and because a defect of muscle development in PLS3 knock-out zebrafish was reported.^7^ If one of the mechanisms underlying osteoporosis due to *PLS3* genetic variants is indeed a decreased mechanosensing, it is conceivable that part of the defect might be overcome by strenuous physical activity. Such findings may be highly relevant for patient management. In addition, other lifestyle factors and genetic factors like a polygenic risk score will be relevant to correlate with disease severity, especially within families. Currently, quality of life questionnaires are being collected in our cohort for future analysis.

In conclusion, in this study of 28 patients from 11 different families with X-linked osteoporosis due to *PLS3* genetic variants, we confirmed that men are more severely affected than women. Furthermore, we showed that initiation of antiresorptive therapy leads to a reduction in vertebral fracture prevalence and an increase in BMD. However, some patients had progression or developed new vertebral fractures partly due to non-compliance, needing anabolic therapy. Close monitoring of patients with DXA scans every 2 y and education of patients to contact their specialist when a fracture occurs or with symptoms suggestive of new vertebral fractures, is important to detect disease progression early. No relation could be shown between the different types of genetic variants and their respective location with multiple clinical endpoints in 72 men, including 18 men from our cohort. More research on the relationship between physical activity, which could potentially overcome a defect in mechanosensing due to pathogenic *PLS3* variants, and the disease course is relevant for patient management. In addition, more research is needed on muscle mass and function, the so far unexplained occurrence of extra skeletal manifestations such as blue sclerae, skin and joint hyperlaxity and neurological manifestations, as well as quality of life questionnaires in patients with *PLS3*-related osteoporosis.

## Supplementary Material

supp_ziaf046

## Data Availability

Sharing raw or processed individualized sequencing results of the patients are not allowed due to General Data Protection Regulation (GDPR). Requests to access the datasets should be directed to M.C.Z., m.c.zillikens@erasmusmc.nl.
