# Supplementary material for: Phenotypic and genetic characteristics of a Dutch cohort of patients with X-linked osteoporosis due to PLS3 genetic variants
Source: JBMR Plus. 2025 Apr 24;9(6):ziaf046. doi: 10.1093/jbmrpl/ziaf046 (PMC12063993; doi:10.1093/jbmrpl/ziaf046)
Supplement: supp_ziaf046 [file supp_ziaf046.pdf]

Supplementary table 1. Genetic and clinical information of patients with *PLS3* genetic variants.

Transcript NM_005032.7	Age [yr]	Sex	Variant	Exon	Functional domain	Type of variant	ACMG Class	Non-vertebral FX	Vertebral FX	Finger / Toe FX	Medication	Previously reported
**Family 1**												
	†	M	c.842del p.(Leu281Trpfs*41)	8	ABD1	Frameshift Predicted to undergo NMD	5	4	5	3	1999-2011 oral BP, 2013-2015 iv BP	
	60	M	c.842del p.(Leu281Trpfs*41)	8	ABD1	Frameshift	5	3	2	0	2011-2016 BP, 2018-to date DMAB	
**Family 2**												
	58	M	c.329C>A p. (Ser110*)	4	Linker	Nonsense Predicted to undergo NMD	4	5	13	0	1994-2000, 2004-2012 oral BP	
**Family 3**												
	50	F	c.343G>A p.(Glu115Lys)	4	Linker	Missense	3	3	0	3	11-2019 start ALN	
	25	M	c.343G>A p.(Glu115Lys)	4	Linker	Missense	3	1	5	0	2015-2020 oral BP	
	23	M	c.343G>A p.(Glu115Lys)	4	Linker	Missense	3	6	4	3	2016-2020 ZOL	

**Family 4**												
	61	M	c.1050_1053del<br>p(Arg350Serfs*17)	10	ABD1	Frameshif<br>t<br>Predicted<br>to<br>undergo<br>NMD	4	4	7	0	2012-2014<br>BP, 2015-<br>2017 TPT,<br>2017-to<br>date<br>DMAB	
	36	F	c.1050_1053del<br>p.(Arg350Serfs*17)	10	ABD1	Frameshif<br>t	4	2	0	0	2022- to<br>date oral<br>PMN	
**Family 5**												
	61	M	c.1097_1101del<br>p.(Asn366Serfs*5)	10	ABD1	Frameshif<br>t<br>Predicted<br>to<br>undergo<br>NMD	4	1	13	1	1988-1995<br>PMN,<br>2011-2017<br>ZOL	1
**Family 6**												
	73	M	c.759_760insAAT<br>p.(Ala253_Leu254insAsn)	8	ABD1	In-frame<br>insertion	3	1	2	0	2005,<br>2007-2010<br>IBN	2
	37	F	c.759_760insAAT<br>p.(Ala253_Leu254insAsn)	8	ABD1	In-frame<br>insertion	3	0	0	0		
**Family 7**												
	80	F	c.235del<br>p.(Tyr79Ilefs*6)	3	EF2	Frameshif<br>t<br>Predicted<br>to<br>undergo<br>NMD	5	1	4	0	2005-<br>02/2024<br>RSN,<br>02/2024-to<br>date RMS	2
	53	M	c.235del<br>p.(Tyr79Ilefs*6)	3	EF2	Frameshif<br>t	5	17	14	0	2008-2009<br>ALN,<br>2009-2010<br>RSN, 2010<br>ZOL	
	57	F	c.235del<br>p.(Tyr79Ilefs*6)	3	EF2	Frameshif<br>t	5	1	0	0	-	

	26	M	c.235del p.(Tyr79Ilefs*6)	3	EF2	Frameshift	5	4	0	1	2005-2010 PMN iv, 2010-2011 ALN	
	30	M	c.235del p.(Tyr79Ilefs*6)	3	EF2	Frameshift	5	4	2	1	2005-2007 PMN iv, 2007-2008 ALN	
	76	F	c.235del p.(Tyr79Ilefs*6)	3	EF2	Frameshift	5	0	4	0	2011-2016 ZOL, 2018-2020 TPT, 2020-2023 ZOL, 12/2023-to date RMS	
	51	F	c.235del p.(Tyr79Ilefs*6)	3	EF2	Frameshift	5	1	0	1	-	
	22	F	c.235del p.(Tyr79Ilefs*6)	3	EF2	Frameshift		4	0	2	-	
	26	M	c.235del p.(Tyr79Ilefs*6)	3	EF2	Frameshift	5	12	3	5	2006-2016 iv BP, 2022 start ZOL	
	28	M	c.235del p.(Tyr79Ilefs*6)	3	EF2	Frameshift	5	13	3	4	2006-2013 PMN iv, 2013-2015 RSN	
**Family 8**											
	48	M	c.1471C>T p.(Gln491*)	13	ABD2	Nonsense Predicted to undergo NMD	5	3	6	1	2011 ALN, 2014- to date RSN	2
	48	M	c.1471C>T p.(Gln491*)	13	ABD2	Nonsense	5	7	3	2	2011-2018 ALN	
**Family 9**											
	50	F	c.1262+1G>A p?	11	ABD2	Splice-site	4	0	0	0	-	

	22	M	c.1262+1G>A<br>p?	11	ABD2	Splice-site	4	28	0	2	2011-2013 PMN, 2016-2019 ZOL	
	17	F	c.1262+1G>A<br>p?	11	ABD2	Splice-site	4	1	0	0	2018-2019 PMN iv	
**Family 10**	†	M	c1178_1180delinsA<br>pLeu393*	10	ABD1	Nonsense	5	8	4	0	07/2014-12/2014 ALN, 2014-2019 RSN	
**Family 11**												
	†	M	c.748+1G→A<br>p.(Glu249_Ala250ins12)	7	ABD1	Insertion	3	6	10	0	1992-2000 PMN, 2001-2006 ALN, 2007-2012 ALN	[2]

1. Costantini A, Krallis P, Kämpe A, et al. A novel frameshift deletion in PLS3 causing severe primary osteoporosis. *J Hum Genet*. Aug 2018;63(8):923-926.
2. van Dijk FS, Zillikens MC, Micha D, et al. PLS3 mutations in X-linked osteoporosis with fractures. *N Engl J Med*. Oct 17 2013;369(16):1529-36.

Supplementary table 2. Intra-familial phenotypic differences of family 7.

	Age at first fracture	Sex	Total number of fractures	Age at first LS BMD measurement	First LS Z-score	Age at last LS BMD measurement	Last LS Z-score	Duration of treatment (years)	Extra-skeletal characteristics	Dairy intake (s/day)	Exercise in childhood	Ca	Vit D
II-1	30	F	5	61	-0,7	79	-2.0 (T-score distal radius)	19	Slight blue sclera, joint hypermobility, skin hyperlaxity	5	N/A*	No	No
II-3	63	F	4	64	-0,9 (T-score)	76	-2.4 (T-score FN)	10	N/A*	3	N/A*	No	Yes
III-1	1	F	2	43	0.5	55	0.9	0	No	3	N/A*	No	Yes
III-4	12	F	2	40	0.0	51	-0.4	0	Blue sclera, joint hypermobility, skin hyperlaxity	6	N/A*	No	Yes

IV-2	6	M	5	8	-1,8	25	-1,3	6	Slight blue sclera, joint hypermobility	4	Baseball for at least 13 years, fitness	No	Yes
IV-3	8	M	7	10	-2,1	29	-2,1	3	Joint hypermobility, skin hyperlaxity	2	Baseball for at least 11 years	No	No
IV-7	7	M	20	18	-4,5	24	-3,7	11	Joint hypermobility, skin hyperlaxity	1	Skate, tennis, gym	Yes	Yes
IV-8	6	M	22	16	-3,7	26	-2,6	9	Joint hypermobility, skin hyperlaxity	2	Karate, swimming	Yes	Yes
IV-9	7	M	6	22	-2.0	-	-	0	Joint hyperlaxity	5	N/A*	No	Yes

LS: lumbar spine; s/day: servings/day; Ca: calcium supplementation; Vit D: vitamin D supplementation; M: male; W: female

*N/A: not available
